# Supplementary material for: On-site growth of perovskite nanocrystal arrays for integrated nanodevices
Source: Nat Commun. 2023 Jul 6;14:3883. doi: 10.1038/s41467-023-39488-0 (PMC10326253; doi:10.1038/s41467-023-39488-0)
Supplement: Supplementary file 1 — Supplementary Information [file 41467_2023_39488_MOESM1_ESM.pdf]

Supplementary Information

On-site growth of perovskite nanocrystal arrays for integrated nanodevices

Authors

Patricia Jastrzebska-Perfect[1,2], Weikun Zhu[2,3], Mayuran Saravanapavanantham[1,2], Zheng Li[1,2], Sarah O. Spector[1,2], Roberto Brenes[1,2], Peter F. Satterthwaite[1,2], Rajeev J. Ram[1,2], Farnaz Niroui[1,2]*

Affiliations

[1]Department of Electrical Engineering and Computer Science, Massachusetts Institute of Technology, Cambridge, MA 02139, USA

[2]Research Laboratory of Electronics, Massachusetts Institute of Technology, Cambridge, MA 02139, USA

[3]Department of Chemical Engineering, Massachusetts Institute of Technology, Cambridge, MA 02139, USA

*Corresponding author. Email: fniroui@mit.edu

This PDF file includes:

Supplementary Figures 1-13
Supplementary Tables 1-2
Supplementary Notes 1-6
Supplementary References

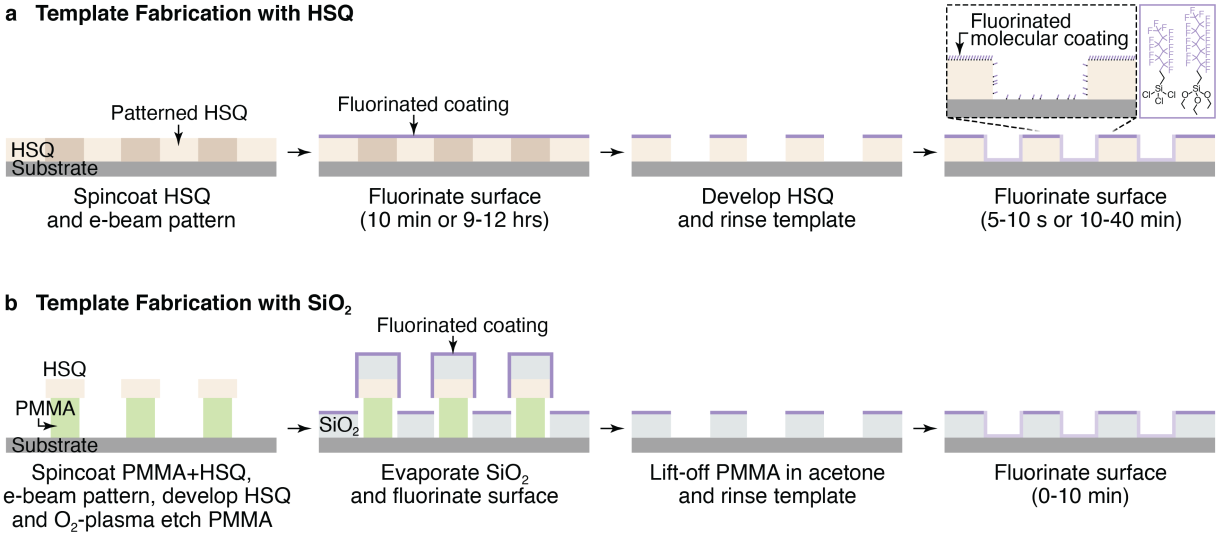

Supplementary Fig. 1 | Template fabrication scheme. a, HSQ-template fabrication. HSQ is spin-coated onto the substrate and exposed using e-beam lithography in the desired pattern. A fluorinated molecule is assembled on the HSQ surface for 10 min (PFTS) or 9-12 hours (PFDTES) in vapor phase in a vacuum desiccator. The resist is developed during which the molecular layer on the unexposed regions is lifted-off. The template is rinsed with DI water and isopropanol, then gently dried using a stream of nitrogen. Finally, the contact angle within the wells is tuned through a second, short vapor phase molecular growth step (5-10 s for PFTS and 10-40 min for PFDTES). **b,** SiO_2-template fabrication. HSQ on PMMA, spin-coated onto the substrate, is exposed and developed, and PMMA is removed from unprotected regions through oxygen plasma etching. SiO_2 is deposited through electron-beam evaporation, and a fluorinated molecule is grown for 9-12 hours (PFDTES). HSQ-PMMA pillars are lifted-off with acetone, then rinsed in isopropanol and gently dried under nitrogen. Finally, the contact angle is tuned through a short molecular growth step (PFDTES for 0-10 min).

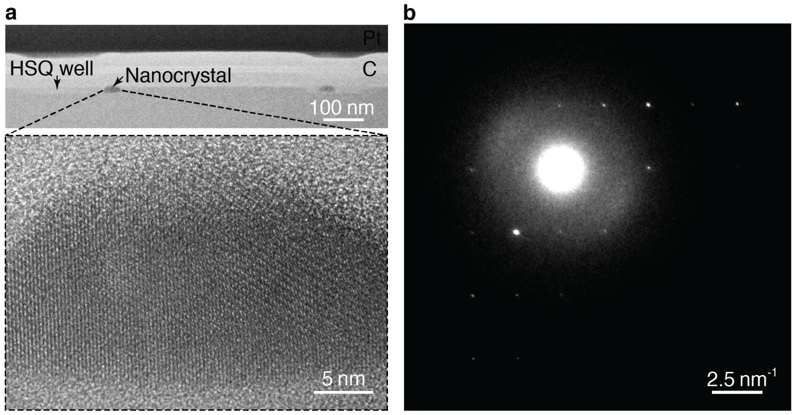

Supplementary Fig. 2 | Nanocrystals are single crystalline. a, TEM image of nanocrystals in a triangular HSQ well cross-sectioned by focused-ion beam milling. **b,** Electron diffraction of the nanocrystal.

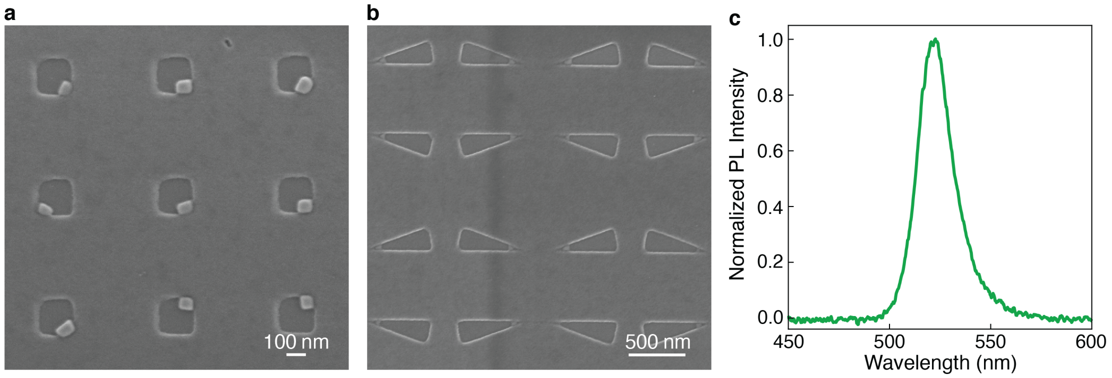

Supplementary Fig. 3 | On-site growth of $CH_3NH_3PbBr_3$ perovskite nanocrystals. SEM images of **a,** square and **b,** triangular HSQ wells containing $CH_3NH_3PbBr_3$ nanocrystals grown given 30° and 45° contact angles, respectively, and precursor concentration of 0.45 M. **c,** Representative photoluminescence (PL) spectrum of $CH_3NH_3PbBr_3$ nanocrystals formed.

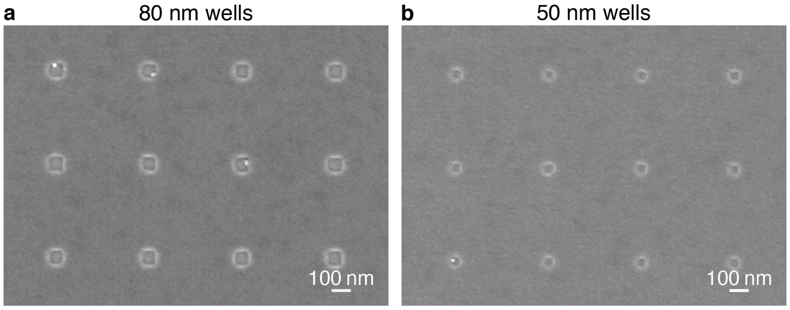

Supplementary Fig. 4 | Lack of well filling prevents nanocrystal formation. Nanocrystals formed in HSQ-templates containing square wells of **a,** 80 nm, or **b,** 50 nm side length, yielding nanocrystals ~ 23 nm and ~ 20 nm in size, respectively. Many empty wells result as they remain unfilled during precursor solution spin-coating due to their small size.

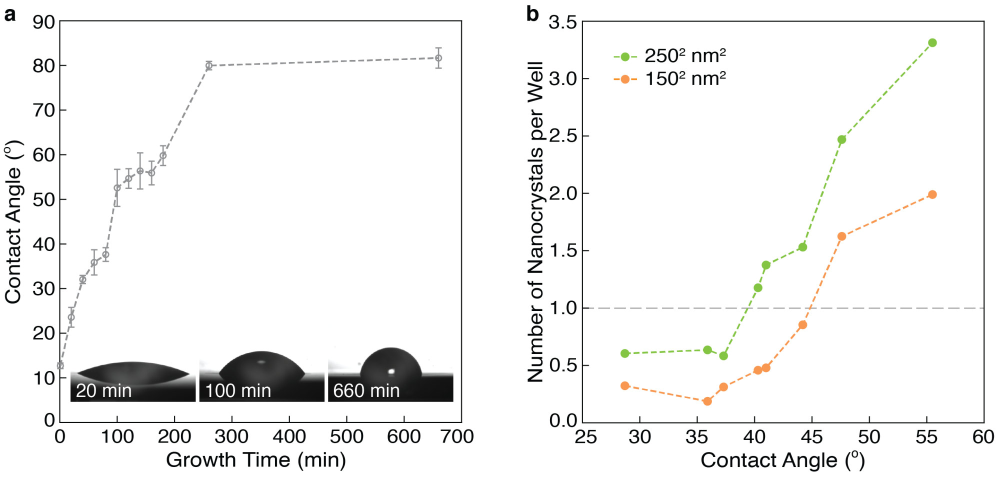

Supplementary Fig. 5 | Contact angle tuning to control nanocrystal formation. a, Contact angle (mean ± standard deviation) of DMSO on PFDTES-coated Si/SiO_2 substrate as a function of PFDTES growth time. Insets show examples of droplet profiles used for determining contact angles. **b,** Number of nanocrystals formed per well as a function of DMSO contact angle tuned by PFDTES growth time for (20°, 80°, 80°) triangle wells of 150^2 nm^2 or 250^2 nm^2 area and 22 nm height. Each data point is a measured average over 96 wells. Note that for larger wells, more nanocrystals per well are observed for equivalent contact angles.

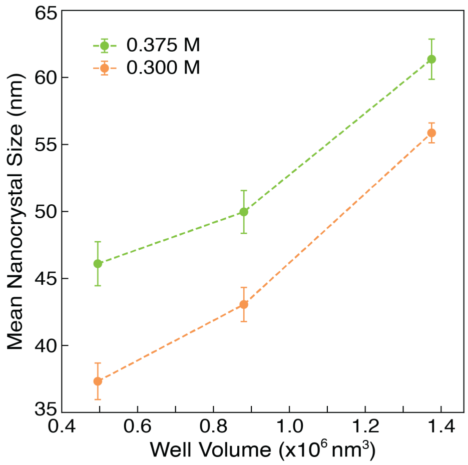

Supplementary Fig. 6 | Increasing nanocrystal size by increasing precursor concentration. The mean ± standard error size of nanocrystals formed, measured for more than 30 single-nucleation wells, 22 nm-thick, as a function of well volume with precursor concentration of 0.300 M and 0.375 M. Here, the nanocrystal size is presented as the average of its lateral dimensions.

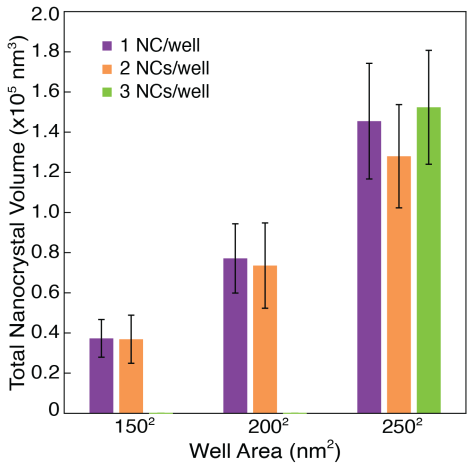

Supplementary Fig. 7 | Increasing nanocrystal size by increasing well size. Nanocrystal (NC) size (mean ± standard deviation), presented as its volume, as a function of increasing well area with a constant well height of 22 nm. Here, the total nanocrystal volume corresponds to the sum of volumes for all nanocrystals contained within a given well. The nanocrystals' lateral dimensions are measured based on SEM images, and their heights using AFM imaging.

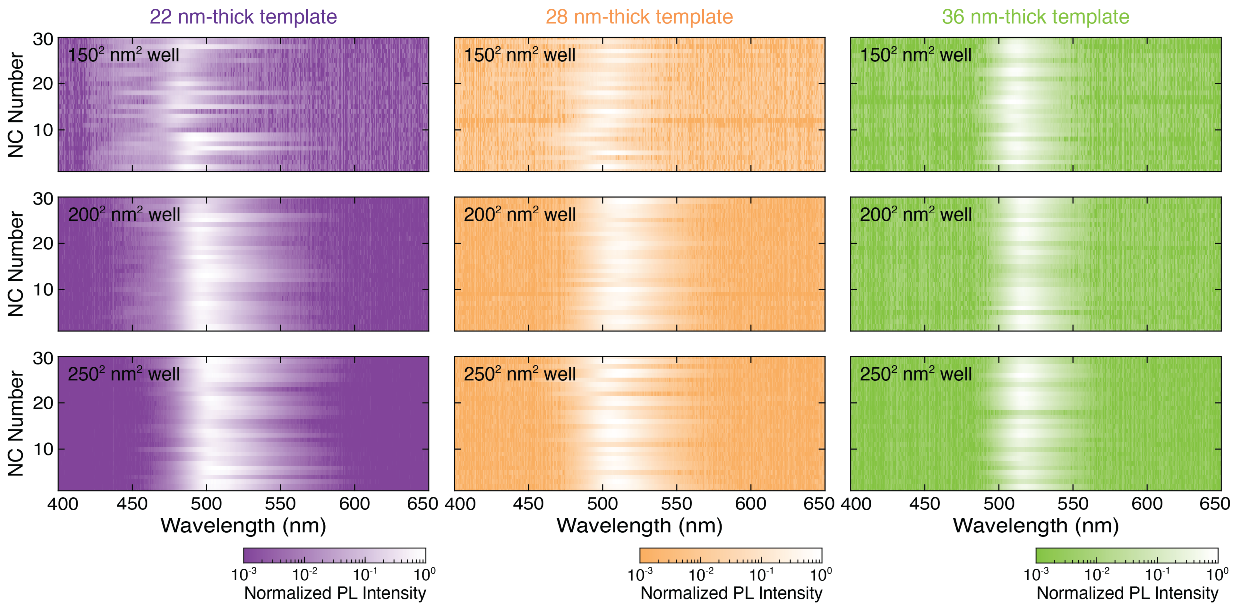

Supplementary Fig. 8 | Photoluminescence (PL) spectra for nanocrystals from wells of increasing area or thickness. PL spectra are normalized by the intensity of the brightest nanocrystal (NC) for each (area, thickness) combination.

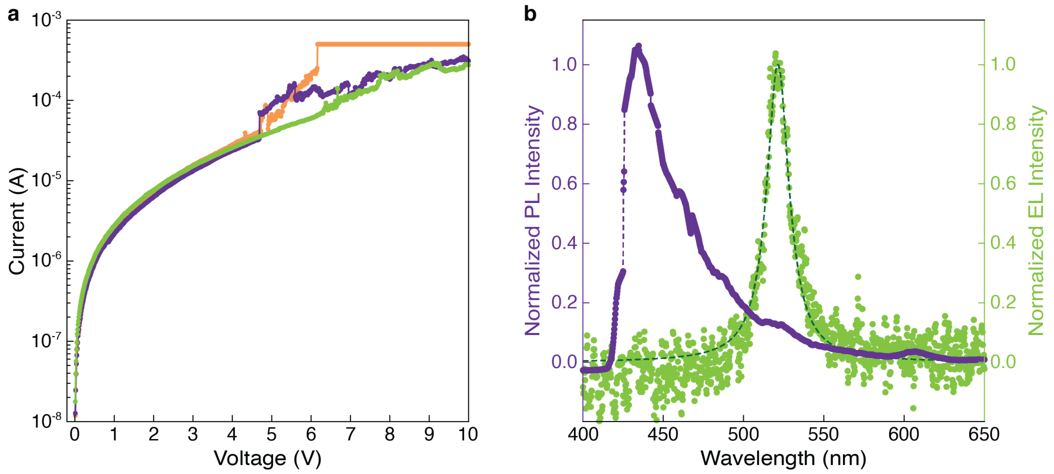

Supplementary Fig. 9 | NanoLED performance. a, Representative current-voltage characteristics for a nanoLED array. **b**, Sample normalized photoluminescence (PL) and electroluminescence (EL) spectra for a nanoLED array. Spectra were collected by passing emission through a dichroic filter (423 nm cut-on wavelength) to remove the excitation source (see Methods). Note that the photoluminescence spectrum includes emission from the TPBi layer, while the EL spectrum does not.

Supplementary Note 1. Critical contact angle

The critical contact angle θ_c refers to the contact angle between the liquid in a well and the well's sidewall below which an arc meniscus exists[1-3]. The critical contact angle can be determined based on Supplementary Fig. 10 in which the liquid forms a contact angle θ with the sidewalls. In this design, an arc meniscus exists if:

$$\frac{\alpha}{2} + \phi + \theta + \frac{\pi}{2} = \pi \tag{1}$$

Since $\phi \geq 0$, the critical contact angle, given by $\phi = 0$, is

$$\theta_c = \frac{\pi}{2} - \frac{\alpha}{2} \tag{2}$$

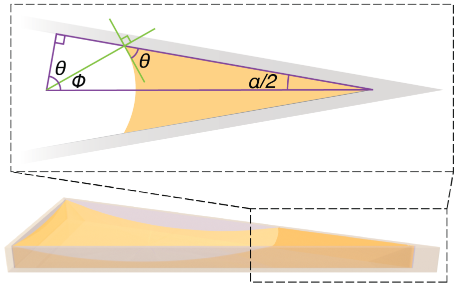

Supplementary Fig. 10 | Critical contact angle calculation. Schematic used in calculating the critical contact angle. Here, θ is the contact angle and α the corner angle.

The critical angle changes with the corner angle α which varies with the well's geometry. Supplementary Table 1 summarizes the critical contact angles evaluated for the four well designs used in the studies of Fig. 2.

Supplementary Table 1 | Critical contact angles for studied well geometries.

Well Geometry	θ_{C1}	θ_{C2}
Square	45°	–
(60°, 60°, 60°) triangle	60°	–
(44°, 68°, 68°) triangle	56° (for 68° corner)	68° (for 44° corner)
(20°, 80°, 80°) triangle	50° (for 80° corner)	80° (for 20° corner)

Supplementary Note 2. Positional accuracy

The positional accuracy A of each well geometry is defined as the expected distance of nanocrystals from a chosen reference point, given by:

$$A = \frac{\sum_{i=1} \sqrt{(x_i - x_c)^2 + (y_i - y_c)^2}}{n} \tag{3}$$

where (x_i, y_i) represents the coordinates of nanocrystal i, (x_c, y_c) represents the coordinates of the reference point, and n is the total number of nanocrystals. Since the ideal well geometry would yield all nanocrystals located within one particular corner, cluster analysis was performed to identify the corner containing the greatest fraction of nanocrystals. The center of this cluster was then set as (x_c, y_c). In this definition, if all nanocrystals were located in the cluster having center (x_c, y_c), then A would approach 0. Therefore, a lower value of A corresponds to a higher degree of accuracy.

As shown in Fig. 2d, a higher positional accuracy is observed for the equilateral triangle than for the square well. To understand this difference, we consider 12 wells of each geometry (i.e. the least common multiple for the two, assuming one nanocrystal per well), whereby each square has length a and each equilateral triangle has length b. Since both geometries are symmetric, we expect the nanocrystals to be evenly distributed across the corners of the wells. The positional accuracy for the square is then:

$$A_{square} = \frac{0 + 3a + 3a\sqrt{2} + 3a}{12} = \frac{a(2 + \sqrt{2})}{4} \tag{4}$$

The positional accuracy of the equilateral triangle is given by:

$$A_{triangle} = \frac{0 + 4b + 4b}{12} = \frac{2b}{3} \tag{5}$$

For the geometries to have the same area, the following condition is imposed:

$$b = \frac{2a}{3^{1/4}} \tag{6}$$

Therefore, the ratio of the positional accuracies $A_{triangle}/A_{square}$ is 1.19, which matches the experimentally observed 1.16 ± 0.08 (mean ± standard deviation).

Supplementary Note 3. Asymmetric meniscus for nanocrystal positioning

Meniscus asymmetry, visualized in Supplementary Fig. 11a, establishes a pressure gradient within the well, which drives nanocrystal movement. The finite thickness of the film between the main terminal meniscus (MTM) and bottom of the well introduces a disjoining pressure[4]. The total pressure at a position x, $P(x)$, is given by:

$$P(x) = P_{bulk} + \frac{A_H}{6\pi[\delta(x)]^3} \tag{7}$$

where P_{bulk} is the bulk pressure in the solution, A_H is the Hamaker constant between the liquid and the underlying surface, and $\delta(x)$ is the height of the thin film above the solid surface. The corresponding force acting on a particle in solution, assuming the absence of diffusion and convection, is then:

$$\mathbf{F} = \oiint (P\,\hat{n})dA \tag{8}$$

where the integral is taken over the surface of a particle, and $\hat{n}$ is the unit normal vector pointing inward. Using the divergence theorem, and assuming that a pressure gradient is acting on a cube with side length l aligned with the gradient, and that the scale of the cube is small relative to that of the gradient, the net force is given by:

$$\mathbf{F} = l^3 \nabla P \tag{9}$$

The work done to move a particle in this field is simply:

$$U(x_1, x_2) = \int_{x_1}^{x_2} F(x)dx = l^3[P(x_1) - P(x_2)] \tag{10}$$

Thus, the energy of the particle at any point in the well relative to its energy when confined within the arc meniscus is:

$$U(x) = \frac{A_H}{6\pi} l^3 \left(\frac{1}{[\delta(x)]^3} - \frac{1}{\delta_{AM}^3} \right) \tag{11}$$

In Supplementary Fig. 11b, the energy of a 10 nm nanocrystal as a function of position within an elliptical meniscus profile is evaluated. The meniscus profile is described by:

$$\delta(x) = -\frac{b}{a}(a^2 - x^2)^{\frac{1}{2}} \tag{12}$$

where a and b are, respectively, the radius and height of the main terminal meniscus. The arc meniscus height δ_{AM} is 22 nm. The Hamaker constant is approximated by $A_H \approx \sqrt{A_{ll}A_{ss}}$, where

A_{ll} and A_{ss} are the Hamaker constants for the self-interaction of the liquid and solid, respectively[5]. The Hamaker constant for the solvent (dimethyl sulfoxide) is $A_{ll} = 6.72 \times 10^{-20}$ J,[6] while that for the surface (SiO_2) is $A_{ss} = 6.5 \times 10^{-20}$ J.[5]

Supplementary Fig. 11b(ii) shows the energy of the particle for each x of the elliptical menisci depicted in Supplementary Fig. 11b(i), while Supplementary Fig. 11c shows the particle's energy at the meniscus minimum as a function of thin-film height $\delta(x)$. The particle has maximum energy at the meniscus minimum. Importantly, the particle's energy at $x = 0$, the meniscus' contact point with the sidewall, still exceeds its energy at the arc meniscus. The arc meniscus is therefore the energetically preferred location of the particle. It also should be noted that as the film height $\delta(x)$ increases, particle energy decreases, and that as the MTM radius increases, $|dU/dx|$ decreases. A meniscus that is deep (i.e. with large b, hence yielding small $\delta(x)$), and laterally confined (i.e. with small a), therefore promotes particle positioning.

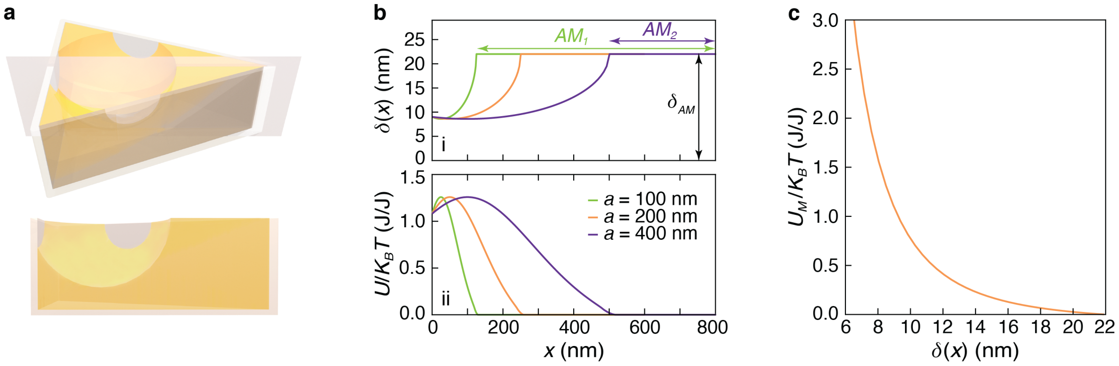

Supplementary Fig. 11 | Pressure gradient force. a, Three-dimensional schematic of a well (top), and its cross-section taken at the defined plane (bottom). **b,** (i) Thin-film height $\delta(x)$ and (ii) normalized nanocrystal energy as a function of position in well, for asymmetric menisci whose main terminal menisci (MTM) have radii a = 100, 200, or 400 nm. **c,** Normalized energy of nanocrystal at meniscus minimum as a function of the thin-film height $\delta(x)$.

Supplementary Note 4. Arc meniscus areas

For the wells studied in Fig. 2, the favorability of positioning relates to arc meniscus area. Compare the arc menisci (AM) yielded by a = 100 nm and a = 400 nm in Supplementary Fig. 11b (i.e. AM_1 and AM_2, respectively). A larger arc meniscus ensures a smaller MTM radius. Therefore, the more solvent the corner angle contains within the arc meniscus during the drying process, the more favorable directional positioning becomes. As a result, the arc meniscus area provides a means for comparing the positioning potential of different corners, and for different contact angles, within the same well geometry.

The arc meniscus areas can be computed by considering Supplementary Fig. 12, where α is the corner angle of the well, θ is the contact angle made by the liquid with the well's sidewalls, and r is the radius of the arc meniscus, provided by Reference [7] and summarized below.

Triangular well, r:

For $0 \le \theta \le \theta_{c_1}$, where A and P are the area and perimeter of the triangle, respectively,

$$r = f_1(G,\theta)P \tag{13}$$

$$f_1(G,\theta) = \frac{2G}{\sqrt{4G(\pi - 3\theta + 3\sin\theta\cos\theta)} + \cos\theta} \tag{14}$$

$$G = \frac{A}{P^2} \tag{15}$$

For $\theta_{c_1} \le \theta \le \theta_{c2}$, where α and β are the angles of the two corners without arc menisci,

$$r = f_3(G,\theta,\alpha,\beta)P \tag{16}$$

$$f_3(G,\theta,\alpha,\beta) = \frac{2G}{\sqrt{4G\left(\frac{\alpha+\beta-2\theta}{2} + \sin\theta\cos\theta + \cos^2\theta\,(\cot\frac{\alpha}{2} + \cot\frac{\beta}{2})\right)} + \cos\theta} \tag{17}$$

Square well, r:

$$r = \frac{A_{eff}}{P_{eff}} \tag{18}$$

$$A_{eff} = A - 4r^2\cos^2\theta + \frac{2\pi - 8\theta}{2\pi}\pi r^2 + 4r^2\sin\theta\cos\theta \tag{19}$$

$$P_{eff} = (P - 8r\cos\theta + 8r\sin\theta)\cos\theta + \frac{2\pi - 8\theta}{2\pi}2\pi r \tag{20}$$

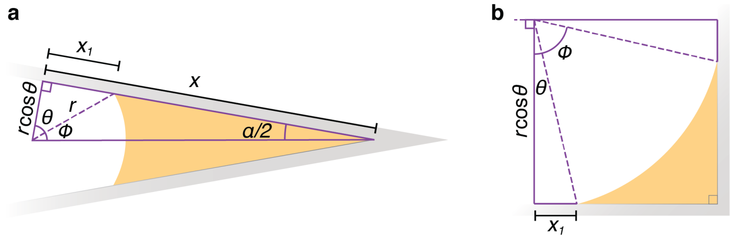

Supplementary Fig. 12 | Arc meniscus. Schematics used in calculating the arc meniscus area for **a,** triangular and **b,** square wells. Here, θ is the contact angle and α the corner angle.

The area of the arc meniscus for the different well geometries used in this study are evaluated below.

Triangular well, area:

We define the area subtended by ϕ as A_1, the area of the triangle of base x_1 as A_2, and the area of the triangle of base x as A_3. The arc meniscus area $A_{AM,tri}$ is then:

$$A_{AM,tri} = 2(A_3 - A_2 - A_1) \tag{21}$$

From Supplementary Fig. 12a,

$$A_1 = \pi r^2 \frac{\phi}{2\pi}$$

$$A_2 = \frac{1}{2} x_1 r \cos\theta$$

$$A_3 = \frac{1}{2} x r \cos\theta$$

$$x_1 = r \cos\theta \tan\theta$$

$$x = \frac{r\cos\theta}{\tan\frac{\alpha}{2}}$$

Therefore, arc meniscus area for each corner of angle α within the triangular well is:

$$A_{AM,tri} = r^2\left(\cos^2\theta\left(\frac{1}{\tan\frac{\alpha}{2}} - \tan\theta\right) - \frac{\pi - \alpha - 2\theta}{2}\right) \tag{22}$$

Square well, area:

We now define the area subtended by ϕ as A_1, the area of each triangle delineated in Supplementary Fig. 12b as A_2, and the area of the square as A_3. The arc meniscus area $A_{AM,sq}$ is then:

$$A_{AM,sq} = A_3 - 2A_2 - A_1 \tag{23}$$

From Supplementary Fig. 12b,

$$A_1 = \pi r^2 \frac{\phi}{2\pi}$$

$$A_2 = \frac{1}{2} x_1 r \cos\theta$$

$$A_3 = r^2 \cos^2\theta$$

$$\phi = \frac{\pi}{2} - 2\theta$$

$$x_1 = r \cos\theta \tan\theta$$

Thus, the area of the arc meniscus for each corner within the square well is:

$$A_{AM,sq} = r^2 \left(\cos^2\theta\,(1 - \tan\theta) - \frac{\pi}{4} + \theta\right) \tag{24}$$

Supplementary Note 5. Nanocrystal photoluminescence quantum yield

The photoluminescence quantum yield (PLQY) of a nanocrystal, Q_s is determined by:

$$\frac{N_s}{N_r} = \frac{A_s Q_s}{A_r Q_r} \tag{25}$$

where N is number of photons emitted per unit time, A is number of photons absorbed per unit time, subscript s refers to the sampled nanocrystal and subscript r refers to the reference.

The reference sample is a thin film of $CsPbBr_3$ quantum dots on a quartz substrate with peak emission of 512 nm. The quantum yield of this sample, Q_r, is determined using an integrating sphere (Labsphere). The number of photons absorbed by this sample each second is given by:

$$A_r = \frac{I}{E_{photon}}(\pi r^2)10^{-Abs} \tag{26}$$

where I is the power of the excitation laser per unit area, E_{photon} is the energy of the exciting photon, r is the radius of the laser spot size, and Abs is the absorbance of the film at the excitation wavelength ($\lambda = 405$ nm), measured via a Perkin Elmer Lambda 1050 UV/VIS/NIR Spectrophotometer with an integrating sphere attachment.

The number of photons absorbed by the nanocrystal each second is given by

$$A_s = \frac{\sigma I}{E_{photon}} \tag{27}$$

where σ is the absorption cross-section of the nanocrystal. The absorption cross-section is estimated using Reference [8], which establishes that $\sigma = 9.45 \times 10^4\,[\mathrm{cm}^{-1}] * V_{NC}[\mathrm{cm}^3]$ where V_{NC} is the volume of the nanocrystal.

The number of photons emitted per unit time is determined by averaging the number of photons detected on an APD over 30 s of integration time. The PLQY is computed for 30 nanocrystals of two well sizes giving 8.71 ± 0.72 % and 16.6 ± 0.38 % for nanocrystals with volumes of 54,000 nm^3 and 102,000 nm^3, respectively. These are formed in 200^2 nm^2 and 250^2 nm^2 wells, 22 nm-thick.

Supplementary Note 6. Single nanoLED electroluminescent power

The electroluminescent power of a single nanoLED is estimated based on segmentation of the LED arrays in Fig. 4e, where each nanoLED is bound by a sector as shown in Supplementary Fig. 13a-b. With the dark level of the image subtracted, the pixel values in the ith sector are summed and converted to the electroluminescent photon number (N_i) as

$$N_i = C \sum_{j \in \mathrm{sector_i}} a_j \tag{28}$$

where a_j is the value of the jth pixel in sector i, and C is a constant representing the electroluminescent photon number per digital count. C is calibrated by exposing the CCD to a collimated 513 nm laser beam with a known power level (photon flux) and fitting the integrated photon number from the laser with the total digital counts on the CCD chip. We find that the calibrated $C \approx 1.1$ photon/count is close to the theoretical value (≈ 1.0 photon/count) based on the full-well depth, the analog gain, and the quantum efficiency of the CCD chip, which justifies our calibration method. The power of the nanoLED i (P_i) can then be estimated as

$$P_i = \frac{N_i \hbar\omega}{t} \tag{29}$$

where $\hbar\omega \approx 2.4$ eV is the averaged electroluminescent photon energy and t is the integration time.

In Supplementary Fig. 13c-d, we present the histograms of the single LED power in two different arrays which give similar average power of ~ 0.6 fW. Note that we have not accounted for the power loss resulting from the collection optics. As a result, the estimation provided here is conservative and serves as a lower bound.

The external quantum efficiency (EQE) of a single nanoLED can be calculated as the ratio of the photon emission rate to the electron injection rate. Assuming all the current we measured passes through our nanoLEDs, the averaged EQE can then be expressed as:

$$\overline{\eta}_{eqe} = \frac{\overline{P}_i}{\hbar\omega} \Big/ \frac{I}{e\,n} \tag{30}$$

where e is the elementary charge, $n = 600$ is the number of nanoLEDs on the given electrically-probed pad, $I \approx 3 \times 10^{-4}$A is the total injection current from Supplementary Fig. 9a, and $\bar{\cdot}$ stands for average. We therefore have $\overline{\eta}_{eqe} \approx 5 \times 10^{-10}$.

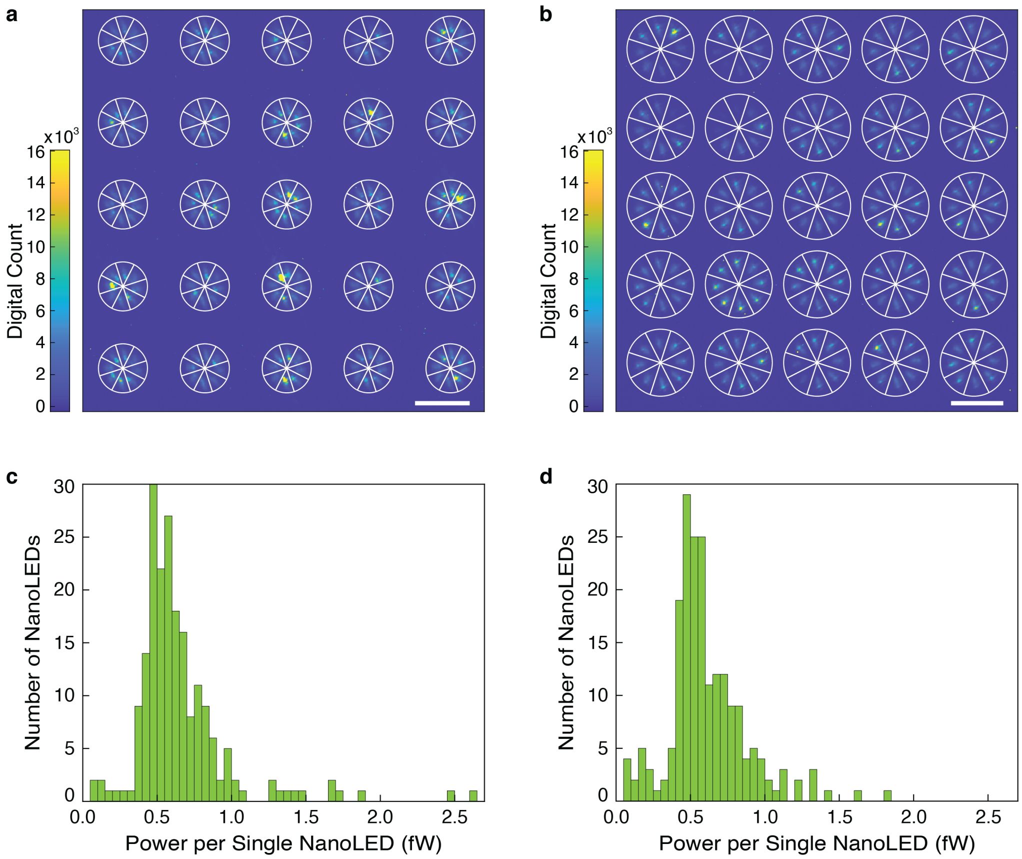

Supplementary Fig. 13 | Estimation of single nanoLED electroluminescent power. a-b, Image segmentation of the two nanoLED arrays in Fig. 4e. **c-d,** Histograms of single LED electroluminescent power based on images in a and b, yielding average power of 0.65 ± 0.33 fW and 0.61 ± 0.27 fW, respectively. The scalebar is 10 µm.

Supplementary Table 2 | Nanocrystal dimensions as a function of well size. The nanocrystal lateral dimensions and height associated with each growth condition presented in Fig. 3b. The dimensions are presented for wells yielding single nucleations. N/A signifies wells that did not yield a measurable fraction of single nucleations. NC length shows the mean lateral dimensions based on the lengths of the major and minor axes of the nanocrystal. Each number represents mean ± standard deviation.

Well height (nm)	Well area (nm^2)	NC major axis length (nm)	NC minor axis length (nm)	NC length (nm)	NC height (nm)	Mean number of NCs/well
22	150^2	42 ± 5	34 ± 4	38 ± 3	21 ± 2	1.2
	200^2	50 ± 5	38 ± 3	44 ± 2	24 ± 2	1.5
	250^2	N/A	N/A	N/A	N/A	2.0
28	150^2	44 ± 8	37 ± 5	40 ± 6	23 ± 2	1.3
	200^2	64 ± 7	42 ± 6	54 ± 6	29 ± 4	1.7
	250^2	72 ± 9	56 ± 5	64 ± 6	36 ± 4	1.8
36	150^2	40 ± 4	31 ± 3	35 ± 3	37 ± 2	1.5
	200^2	N/A	N/A	N/A	N/A	2.1
	250^2	N/A	N/A	N/A	N/A	2.3

References

1. P. Concus, R. Finn, On Capillary Free Surfaces in the Absence of Gravity. *Acta Math.* **132**, 174–198 (1974).
2. R. Finn, Existence Criteria for Capillary Free Surfaces without Gravity. *Indiana Univ. Math. J.* **32**, 439–460 (1983).
3. S. Son, L. Chen, Q. Kang, D. Derome, J. Carmeliet, Contact Angle Effects on Pore and Corner Arc Menisci in Polygonal Capillary Tubes Studied with the Pseudopotential Multiphase Lattice Boltzmann Model. *Computation*. **4**, 1–18 (2016).
4. V. P. Carey, A. P. Wemhoff, Disjoining Pressure Effects in Ultra-thin Liquid Films in Micropassages - Comparison of Thermodynamic Theory with Predictions of Molecular Dynamics Simulations. *J. Heat Transfer*. **128**, 1276–1284 (2006).
5. J. N. Israelachvili, *Intermolecular and Surface Forces* (Academic Press, 2011).
6. H. Takagishi, T. Masuda, T. Shimoda, R. Maezono, K. Hongo, Method for the Calculation of the Hamaker Constants of Organic Materials by the Lifshitz Macroscopic Approach with Density Functional Theory. *J. Phys. Chem. A*. **123**, 8726–8733 (2019).
7. P. Jia, M. Dong, L. Dai, Threshold Pressure in Arbitrary Triangular Tubes using RSG Concept for All Wetting Conditions. *Colloids Surf. A: Physicochem. Eng. Asp*. **302**, 88–95 (2007).
8. F. Zhang, Y. Liu, S. Wei, J. Chen, Y. Zhou, R. He, T. Pullerits, K. Zheng, Microscopic Morphology Independence in Linear Absorption Cross-section of $CsPbBr_3$ Nanocrystals. *Sci. China Mater*. **64**, 1418–1426 (2021).
